# Exercise intensity modulates brachial artery retrograde blood flow and shear rate during leg cycling in hypoxia

**DOI:** 10.14814/phy2.12423

**Published:** 2015-06-02

**Authors:** Erika Iwamoto, Keisho Katayama, Koji Ishida

**Affiliations:** 1 School of Health Sciences, Sapporo Medical UniversitySapporo, Japan; 2 Research Center of Health, Physical Fitness and Sports, Nagoya UniversityNagoya, Japan; 3 Graduate School of Medicine, Nagoya UniversityNagoya, Japan

**Keywords:** Aerobic exercise, exercise intensity, hypoxia, retrograde shear rate

## Abstract

The purpose of this study was to elucidate the effect of exercise intensity on retrograde blood flow and shear rate (SR) in an inactive limb during exercise under normoxic and hypoxic conditions. The subjects performed two maximal exercise tests on a semi-recumbent cycle ergometer to estimate peak oxygen uptake (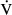
O_2peak_) while breathing normoxic (inspired oxygen fraction [FIO_2_ = 0.21]) and hypoxic (FIO_2_ = 0.12 or 0.13) gas mixtures. Subjects then performed four exercise bouts at the same relative intensities (30 and 60% 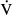
O_2peak_) for 30 min under normoxic or hypoxic conditions. Brachial artery diameter and blood velocity were simultaneously recorded, using Doppler ultrasonography. Retrograde SR was enhanced with increasing exercise intensity under both conditions at 10 min of exercise. Thereafter, retrograde blood flow and SR in normoxia returned to pre-exercise levels, with no significant differences between the two exercise intensities. In contrast, retrograde blood flow and SR in hypoxia remained significantly elevated above baseline and was significantly greater at 60% than at 30% 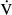
O_2peak_. We conclude that differences in exercise intensity affect brachial artery retrograde blood flow and SR during prolonged exercise under hypoxic conditions.

## Introduction

Aerobic exercise induces increases in blood flow and shear stress in the inactive limbs (Green et al. 2004; Green 2009). Blood flow patterns (oscillatory, antegrade, and retrograde blood flow) also change in the inactive limbs during aerobic exercise, and it has been theorized that changes in blood flow and shear rate (SR) have pivotal implications for aerobic exercise-induced adaptation of vascular function (Green et al. 2011). Exercise-induced enhancement of antegrade SR has been reported to upregulate endothelial vascular function (Tinken et al. 2009; Green et al. 2011), whereas retrograde SR may present a detrimental stimulus to endothelium (Thijssen et al. 2009a, 2014; Schreuder et al. 2014). Accordingly, evaluation of changes in antegrade and retrograde blood flow and SR patterns during exercise may be important to understanding vascular adaptations.

Increased sympathetically mediated vasoconstriction could be the mechanism for augmenting retrograde blood flow and SR in the inactive limb, although the mechanisms underlying oscillatory shear profiles are not fully understood (Padilla et al. 2010; Casey et al. 2012; Thijssen et al. 2014). Sympathetic-vasoconstriction outflow during aerobic exercise is affected by a number of factors, including intensity of exercise and inspired oxygen fraction (FIO_2_). Under normoxic condition, sympathetic vasomotor outflow during dynamic exercise decreases or does not change from that at rest if the exercise intensity is light or mild, and gradually rises thereafter in proportion to the increase in exercise intensity (Saito et al. 1993; Callister et al. 1994; Ichinose et al. 2008; Katayama et al. 2014a). In contrast, sympathetic nervous activity is enhanced during dynamic leg exercise under hypoxic conditions, even if the exercise intensity is low (Katayama et al. 2011). Exercise is performed under hypoxic conditions in many situations; for example, at high altitude or under pathophysiological conditions. Aerobic exercise training is typically constant-load exercise. In addition, exercise intensity is clearly important to an exercise program and affects endothelial adaptation (Goto et al. 2003; Harris et al. 2008; Hallmark et al. 2014). However, the effect of exercise intensity on retrograde SR during constant-load exercise in hypoxia is unclear.

Increases in exercise intensity affect not only on muscle vasomotor outflow, but also cutaneous thermoregulatory responses. At the onset of exercise, retrograde SR is enhanced, at least in part because of increased sympathetic vasoconstriction. However, when exercise intensity and duration are sufficient to induce thermoregulatory vasodilation, retrograde SR that has increased with increasing exercise intensity is decreased by cutaneous vasodilation (Kenney and Johnson 1992; Simmons et al. 2011a). Thus, it is possible that retrograde SR in the inactive limb during prolonged exercise in normoxia is not affected by exercise intensity. In contrast, hypoxia potentiates exercise-induced sympathetic neural activation. Enhanced retrograde SR remains elevated above baseline values during 30 min of exercise in hypoxia, even if thermoregulatory responses occur (Iwamoto et al. 2013). From this we hypothesized that cutaneous vasodilation is not a major determinant of retrograde SR during prolonged hypoxic exercise, and that the enhancement of retrograde SR caused by an increase in exercise intensity would therefore persist during 30 min of exercise under hypoxic conditions.

The purpose of the present study was to elucidate whether the blood flow and SR patterns in an inactive limb during exercise in normoxia and hypoxia vary in response to different exercise intensities. We observed blood flow and SR patterns in a brachial artery during leg cycling exercise at light (30% 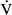
O_2peak_) and moderate (60% 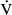
O_2peak_) intensities while subjects breathed normoxic and hypoxic gas mixtures.

## Methods

### Subjects

Eight healthy males [age, 22.2 ± 0.8 years; height, 173.6 ± 2.7 cm; body mass, 69.1 ± 2.9 kg, mean values ± standard error (SE)] with no history of cardiorespiratory disease participated in this study. Subjects were informed of the experimental procedures and possible risks involved, and their informed consent was obtained. This study was approved by the Human Research Committee of the Research Center of Health, Physical Fitness and Sports at Nagoya University.

### Experimental procedures

During a preliminary visit to the laboratory, subjects were familiarized with the equipment to be used. The air in the laboratory was maintained at a temperature of 22–23°C. During practice sessions, subjects were instructed to extend both arms laterally and placed on the stands, and were shown how to hold their arms during leg cycling. Subjects performed leg cycling, using an electromechanically braked ergometer (Aerobike 75XL III, Combi, Tokyo, Japan) in a semi-recumbent position (Saito et al. 1993, 1997).

Subjects were required to visit the laboratory at the same time of day for six additional days. Subjects were asked to abstain from caffeinated beverages and to avoid strenuous exercise for 12 h before the experiment. The study was performed at least 3 h after a light meal. During the first and second sessions, maximal exercise tests were performed with subjects breathing normoxic (FIO_2_ = 0.21) and hypoxic (FIO_2_ = 0.12 or 0.13) gas mixtures. Normoxic and hypoxic gases were provided by a gas generator (YHS-310, YKS, Nara, Japan). In the present study, the lower limit of arterial oxygen saturation (SpO_2_) during the maximal exercise test was set at 70%. During the test, one subject developed SpO_2_ below 70% while breathing a hypoxic gas mixture (FIO_2_ = 0.12). Therefore, we used another gas mixture (FIO_2_ = 0.13) for this subject. The maximal exercise test began at an initial power output of 90 W, and the workload was increased by 15 W every 1 min until the subject was exhausted. The pedaling rate was held constant at 60 rpm with the aid of a metronome, and exhaustion was defined as the point at which the subject could not maintain the pedal cadence >55 for five consecutive revolutions. Oxygen uptake (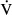
O_2_), SpO_2_, and heart rate (HR) were recorded during the maximal exercise test and were averaged every 30 sec afterward. The highest value of 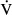
O_2_ was used as the peak oxygen uptake (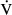
O_2peak_). The order of exercise in normoxia and hypoxia was randomized, and subjects were blinded to the gas mixtures given.

On the third and sixth occasions, submaximal exercise tests were performed. After a 30-min rest, subjects moved to the cycle ergometer, where they wore a facemask attached to a gas generator. Initially, subjects rested for 5 min under normoxia (FIO_2_ = 0.21; Rest 1). After Rest 1, the inspiratory gas mixture was either maintained (FIO_2_ = 0.21) or switched to a hypoxic (FIO_2_ = 0.12 or 0.13) gas mixture. Subjects were exposed to this gas mixture for 10 min at rest before exercise (Rest 2). They then performed submaximal exercise at the same relative exercise intensities (light 30% 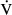
O_2peak_ and moderate 60% 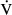
O_2peak_) for 30 min while subjects breathed normoxic (FIO_2_ = 0.21) or hypoxic (FIO_2_ = 0.12 or 0.13) gas mixtures. Subjects were asked to report their rate of perceived exertion (RPE) just before the end of the 30-min exercise bout (Borg 1982). The trials were performed in a random order, and the subjects were blinded to the gas mixture.

### Measurements

#### Respiratory and cardiovascular variables

Respiratory variables during exercise were determined using an online system for breath-by-breath measurement (Katayama et al. 2011, 2012; Iwamoto et al. 2012, 2013). The gas fractions were analyzed using a mass spectrometer (ARCO-1000, Arco System, Chiba, Japan) that was calibrated and confirmed before each test. Expired gas volume was measured using a Fleisch pneumotachometer (PN-230, Arco System, Chiba, Japan). Breath-by-breath data were analyzed continuously using customized computer software to calculate 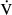
O_2_ and expiratory minute ventilation (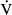
E). SpO_2_ was measured with a pulse oximeter (Biox 3740, Datex-Ohmeda Inc., Madison, WI) placed on the tip of the right forefinger.

A three-lead electrocardiogram (ECG AB-621, Nihon Koden, Tokyo, Japan) was performed and HR was calculated from the R-R interval. Systolic and diastolic arterial blood pressure (BP) was measured in the left arm using an automated BP unit (STBP-780, Colin Medical Instruments, San Antonio, TX). Before taking any measurements, pressure value functions were confirmed by means of a mercury sphygmomanometer, which was joined to the pressure line using a Y-connector (Tanaka et al. 1996). Mean arterial pressure (MAP) was calculated using the following equation: MAP = ([SBP – DBP]/3) + DBP. The Korotkoff sound was amplified using the monitor, and a stereo headset worn by the experimenter allowed for a manual confirmation of the algorithm-determined BP values.

ECG and SpO_2_ signals were sampled at a frequency of 1000 Hz through an analog-to-digital converter (Power Lab 16/s, ADInstruments, Bella Vista, NSW, Australia) and stored in a computer (MT7000, EPSON, Nagano, Japan). The respiratory and cardiovascular variables at rest were averaged over the last 1 min of Rest 1 and Rest 2. During exercise, parameters were averaged over a 30-sec sampling interval.

#### Skin blood flow and myoelectrical activity

Skin blood flow in the right forearm (mL·min^−1^·100 g^−1^) was measured by laser blood flowmetry (FLO-C1, Omegawave Inc., Tokyo, Japan). Skin vascular conductance (mL·min^−1^·100 g^−1^·mmHg^−1^) was calculated as the ratio of skin blood flow to MAP. Electromyography (EMG) with active electrodes was used to verify a lack of muscular movement in the radial and ulnar muscles (brachioradialis and flexor carpi ulnaris muscles, respectively) of the right forearm (Tanaka et al. 2006; Iwamoto et al. 2012, 2013). The skin surface was cleaned with alcohol and rubbed with sand particles. Surface bipolar electrodes (Ag–AgCl, 6-mm contact diameter, 10-mm inter-electrode distance) were placed on the muscle bellies. The EMG signals were connected to an amplifier (input impedance 5.1 MΩ, gain 1000×, common-mode rejection ratio >110 dB), with a bandwidth of 5 to 500 Hz (FA-DL-140 sensor and FA-DL-720 8-main amplifier unit, four assist, Tokyo, Japan). Skin blood flow and EMG signals were sampled and analyzed using a method similar to that for the ECG and SpO_2_ signals. To determine arm muscle activity, the EMG root mean squares (RMS) for the brachioradialis and flexor carpi ulnaris muscles were calculated using commercially available software (Chart version 5, ADInstruments).

#### Brachial blood flow

We measured the right brachial artery blood flow using a high-resolution ultrasound machine (Logiq e, GE Yokogawa Medical Systems, Tokyo, Japan), as reported previously (Iwamoto et al. 2012, 2013). An 8.8-MHz multi-frequency linear probe attached to the ultrasound machine was utilized in the distal third of the arm for simultaneous monitoring of brachial artery diameter and blood velocity. The location of the transducer was marked on the skin to ensure constant placement throughout the study. The ultrasound parameters were set to optimize longitudinal B-mode images of the lumen-arterial wall interface and Doppler velocity (PW-mode). Doppler flow signals were corrected at an insonation angle of 60 degrees and the sample volume gate was set to the full width of the vessel to ensure complete insonation. Images of the brachial artery and associated velocity waveform from the Doppler ultrasound machine (640 × 480 pixels) were stored in a computer at a frequency of 20–30 Hz (Green et al. 2002), using a frame grabber (PXC200AF, CyberOptics, Wilsonville, OR), for offline manual analysis.

Artery diameter (cm) and time-averaged mean blood velocity (cm·s^−1^) were calculated manually using image-analysis software (ImageJ, US National Institutes of Health, Bethesda, MD) (Uehata et al. 1997; Katayama et al. 2010; Iwamoto et al. 2012, 2013). Systolic and diastolic diameters were measured about every 2 sec (average of three points each), and the values were averaged within every 10 sec (Iwamoto et al. 2012, 2013). The Doppler waveform detected by the ultrasound auto program was traced using the ImageJ software to calculate the mean blood velocity. The time-averaged positive and negative mean blood velocities were obtained separately to provide a global index of the velocities of antegrade (positive) and retrograde (negative) blood flows. From the cross-sectional area of the brachial artery (cm^2^) and the time-averaged mean blood velocity (cm·s^−1^), the time-averaged blood flow (mL·min^−1^) was calculated as blood flow = *π *× (diameter/2)^2^ × blood velocity × 60 (Padilla et al. 2010; Simmons et al. 2011a). Antegrade blood flow was calculated by antegrade blood velocity and systolic diameter, and retrograde blood flow was derived from retrograde blood velocity and diastolic diameter (Simmons et al. 2011a; Iwamoto et al. 2012, 2013). SR (s^−1^) was defined as 4 × blood velocity/diameter. Antegrade and retrograde blood velocities and systolic and diastolic diameters were used to calculate antegrade and retrograde SRs (Simmons et al. 2011a; Iwamoto et al. 2012, 2013). Oscillatory shear index, an indicator of the magnitude of shear oscillation, was defined as follows: (¦retrograde SR¦) / (¦antegrade SR¦ + ¦retrograde SR¦). Blood flow parameters at rest were averaged over the last 1 min of each session (Rest 1 and Rest 2), and variables during exercise were averaged over the last 30 sec of every 10 min.

### Statistical analysis

All values are expressed as means ± SE. The assumption of normal distribution was verified using a Kolmogorov–Smirnov test, and the data were normally distributed. Comparisons of cardiorespiratory parameters and workload at exhaustion between normoxia and hypoxia were performed using the paired *t*-test. To determine the behavior of observed variables during exercise at light (30% 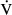
O_2peak_) and moderate (60% 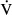
O_2peak_) intensities under each oxygen condition, a three-way ANOVA with repeated measures was performed (intensity × FIO_2_ × time). If the three-way interaction was significant, we tested the simple interaction effects (intensity × FIO_2_) at each time point, (intensity × time) for each oxygen condition, and (FIO_2_ × time) at each exercise intensity. Significant simple interaction effects were followed by an analysis of the simple main effects for each combination of second and third factor levels. If the three-way interaction was not significant, any significant two-way interactions were followed by an analysis of simple main effects at each level of the other factor. If the simple main effects for time were significant, Dunnett's test was used to evaluate differences between baseline data (Rest 1) and each exercise time point. Pearson correlations were used to examine the relationships between changes in skin blood flow and retrograde SR from 10 to 30 min of exercise. The SPSS statistical package (11.5, SPSS Inc., Chicago, IL) was utilized for these analyses. Statistical significance was set at *P *<* *0.05.

## Results

### Maximal exercise test

Cardiorespiratory parameters and workload at exhaustion during maximal exercise testing are shown in Table1. The change in FIO_2_ from a normoxic to a hypoxic condition caused significant reductions in 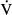
O_2_, SpO_2_, and workload at exhaustion.

**Table 1:** Cardiorespiratory parameters and workload at exhaustion under normoxic and hypoxic conditions.

	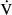 E (L·min^−1^)	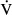 O_2_ (L·min^−1^)	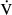 O_2_/BM (mL·kg^−1^·min^−1^)	HR (bpm)	SpO_2_ (%)	Workload (watts)
Normoxia	129.6 ± 13.7	3.2 ± 1.3	46.5 ± 2.2	190.6 ± 1.4	95.8 ± 0.7	263.8 ± 9.1
Hypoxia	124.1 ± 11.3	2.4 ± 1.0*	34.6 ± 1.7*	186.0 ± 1.9	72.3 ± 2.7*	213.8 ± 6.8*

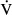
E, expired minute ventilation

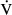
O_2_, oxygen uptake

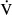
O_2_/BM, oxygen uptake per body weight

HR, heart rate

SpO_2_, arterial oxygen saturation.

Values expressed as mean ± SE.

* *P *<* *0.05 versus Normoxia.

### Submaximal exercise test

#### Baseline descriptive data

Absolute workloads at 30% 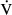
O_2peak_ were significantly lower than at 60% 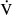
O_2peak_ under both normoxia and hypoxia, and workload at each exercise intensity was higher in normoxia than in hypoxia (normoxia; 30% 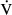
O_2peak_, 56.3 ± 6.8 W, 60% 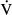
O_2peak_, 144.6 ± 6.7 W. hypoxia; 30% 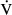
O_2peak_, 41.3 ± 6.6 W, 60% 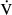
O_2peak_, 110.8 ± 5.0 W). The RMS for both the brachioradialis and flexor carpi ulnaris muscles during 30 min of leg cycling exercise showed no significant differences from Rest 1 in all experiments.

#### Respiratory and cardiovascular variables

Respiratory and cardiovascular variables are shown in Tables2, 3 and Fig. 2. Three-way interaction (intensity × FIO_2_ × time) was significant for HR but not for SpO_2_, SBP, and DBP. For HR, there were significant simple interactions, for intensity × FIO_2_ at 30 min, and for intensity × time under both exercise intensities. For BP, main effects of intensity and time on SBP and DBP were significant, but no other main effects were observed. SBP at 60% 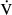
O_2peak_ was significantly higher than at 30% 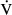
O_2peak_ under both oxygen conditions, but no significant differences were observed between normoxia and hypoxia. There were no significant differences in DBP between 30 and 60% 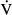
O_2peak_ under normoxic and hypoxic conditions. DBP in normoxia was unchanged from resting levels except at 30 min of exercise at 30% 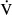
O_2peak_. In contrast, DBP in hypoxic trials showed significant decreases from baseline values as exercise progressed, and DBPs at 30 min in hypoxia at both exercise intensities were lower than those in normoxia. For SpO_2_, there was a significant interaction effect (FIO_2_ × time), and significant main effects of FIO_2_ and time. Hypoxia decreased SpO_2_ at both exercise intensities. RPE at 30 min of exercise at 30% 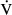
O_2peak_ was significantly lower than at 60% 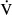
O_2peak_ under normoxic and hypoxic conditions (30% 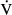
O_2peak_ vs. 60% 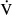
O_2peak_, normoxia; 10.3 ± 0.5 vs. 15.9 ± 0.4. Hypoxia; 9.9 ± 0.9 vs. 15.0 ± 0.3).

**Table 2:** Brachial blood flow, skin blood flow, skin vascular conductance, and SpO_2_ during experiment.

			Exercise			3-way interaction
	Rest 1	Rest 2	10 min	20 min	30 min	*P* value
Antegrade blood flow (mL·min^−1^)						
Norm 30	61.2 ± 7.8	55.5 ± 6.0	90.6 ± 8.0#	112.9 ± 6.5#	127.8 ± 6.3#	*P *<* *0.01
Norm 60	56.6 ± 5.5	59.6 ± 3.9	167.1 ± 11.4 *,#	316.3 ± 28.8*,#	403.4 ± 44.0 *,#	
Hypo 30	60.1 ± 6.8	60.4 ± 3.1	97.8 ± 6.0#	123.6 ± 8.9#	139.9 ± 12.6#	
Hypo 60	75.1 ± 10.0	70.8 ± 7.8	164.1 ± 18.2§,#	270.2 ± 21.5§,#	315.2 ± 22.2§,‡,#	
Retrograde blood flow (mL·min^−1^)						
Norm 30	9.2 ± 0.8	6.6 ± 0.9	22.7 ± 6.8#	26.4 ± 6.7#	21.2 ± 5.8	*P *<* *0.01
Norm 60	9.0 ± 1.3	7.9 ± 1.2	41.0 ± 10.3#	22.2 ± 8.3	15.4 ± 7.5	
Hypo 30	11.7 ± 2.6	15.6 ± 2.5†	46.9 ± 7.0†,#	49.2 ± 8.8†,#	39.8 ± 8.9†,#	
Hypo 60	9.6 ± 2.3	14.0 ± 2.0‡	70.7 ± 8.3§,‡,#	62.5 ± 12.1‡,#	64.0 ± 12.3§,‡,#	
Skin blood flow (mL·min^−1^·100 g^−1^)						
Norm 30	2.2 ± 0.3	2.5 ± 0.3	4.1 ± 0.8	6.2 ± 0.8#	8.4 ± 1.8#	*P *=* *0.41
Norm 60	1.7 ± 0.3	2.0 ± 0.4	7.1 ± 1.7#	13.9 ± 2.1*,#	14.2 ± 2.3#	
Hypo 30	1.7 ± 0.3	1.7 ± 0.2	1.8 ± 0.3	2.7 ± 0.6	2.8 ± 0.7†,#	
Hypo 60	1.7 ± 0.2	1.9 ± 0.2	5.3 ± 1.5	12.5 ± 1.4§,#	12.5 ± 1.3§,#	
Skin vascular conductance (mL·min^−1^·100 g^−1^·mmHg^−1^)						
Norm 30	2.6 ± 0.3	2.8 ± 0.3	4.4 ± 0.8	6.7 ± 0.8#	10.0 ± 2.1#	*P *=* *0.17
Norm 60	2.1 ± 0.4	2.2 ± 0.4	6.4 ± 1.5#	12.6 ± 2.0#	13.0 ± 2.0#	
Hypo 30	1.9 ± 0.3	1.9 ± 0.3	2.1 ± 0.3	3.1 ± 0.7#	3.2 ± 0.8†,#	
Hypo 60	2.0 ± 0.2	2.3 ± 0.4	5.0 ± 1.5	12.6 ± 1.6§,#	12.7 ± 1.5§,#	
SpO_2_ (%)						
Norm 30	97.7 ± 0.2	97.8 ± 0.2	97.9 ± 0.2	97.5 ± 0.2	97.5 ± 0.1	*P *=* *0.54
Norm 60	98.1 ± 0.3	98.1 ± 0.3	97.1 ± 0.4*,#	96.8 ± 0.3#	96.6 ± 0.4#	
Hypo 30	97.7 ± 0.3	86.5 ± 1.7†,#	73.7 ± 1.4†,#	73.8 ± 1.1†,#	72.5 ± 1.5†,#	
Hypo 60	97.7 ± 0.2	84.8 ± 0.9‡,#	70.0 ± 1.6‡,#	69.8 ± 1.8‡,#	69.9 ± 1.5‡,#	

Values expressed as mean ± SE. SpO_2_, arterial oxygen saturation; Norm 30, 30% 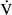
O_2peak_ in normoxia; Norm 60, 60% 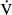
O_2peak_ in normoxia; Hypo 30, 30% 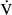
O_2peak_ in hypoxia; Hypo 60, 60% 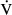
O_2peak_ in hypoxia.

* *P *<* *0.05 Norm 30 versus Norm 60

§ *P *<* *0.05 Hypo 30 versus Hypo 60

† *P *<* *0.05 Norm 30 versus Hypo 30

‡ *P *<* *0.05 Norm 60 versus Hypo 60

# *P *<* *0.05 versus Rest 1 in each trial.

#### Skin blood flow and skin vascular conductance

Three-way ANOVA revealed that there was no significant three-way interaction for skin blood flow and skin vascular conductance (Table2). Significant interactions (FIO_2_ × time and intensity × time) were observed on skin blood flow and skin vascular conductance, but intensity × FIO_2_ was not significant. For skin blood flow, there was a significant main effect of intensity under both normoxia and hypoxia. Skin blood flow during moderate-intensity exercise at 20 min under normoxia and at 20 and 30 min under hypoxia were significantly higher than during light-intensity exercise. In contrast, there was a significant main effect of intensity on skin vascular conductance in hypoxia (*F*_1,7_ = 38.96), but not in normoxia (*F*_1,7_ = 3.21). At 30 min of hypoxic exercise, skin vascular conductance at 30% 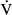
O_2peak_ was significantly lower than at 60% 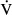
O_2peak_. The main effect of FIO_2_ on skin blood flow and skin vascular conductance was significant only at 30% 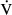
O_2peak_. Skin blood flow and skin vascular conductance at 30 min of light-intensity exercise were significantly lower under hypoxic conditions than under normoxic conditions.

**Table 3:** Blood velocity and diameter under normoxic and hypoxic conditions.

			Exercise			3-way interaction
	Rest 1	Rest 2	10 min	20 min	30 min	*P* value
Antegrade blood velocity (cm·s^−1^)						
Norm 30	7.1 ± 0.8	6.5 ± 0.8	11.2 ± 1.1#	13.4 ± 0.9#	15.2 ± 1.1#	*P *<* *0.01
Norm 60	6.6 ± 0.6	7.0 ± 0.5	22.0 ± 1.5*,#	35.6 ± 2.9*,#	42.0 ± 3.2*,#	
Hypo 30	6.7 ± 0.7	7.0 ± 0.7	11.3 ± 0.9#	13.6 ± 1.6#	15.2 ± 1.6#	
Hypo 60	8.8 ± 1.1	8.2 ± 0.9	20.5 ± 1.8§,#	31.0 ± 1.6§,#	34.6 ± 2.0§,‡,#	
Retrograde blood velocity (cm·s^−1^)						
Norm 30	1.1 ± 0.1	0.8 ± 0.1	2.8 ± 0.9#	3.1 ± 0.8#	2.5 ± 0.7	*P *=* *0.03
Norm 60	1.1 ± 0.2	0.9 ± 0.2	5.5 ± 1.5*,#	2.8 ± 1.2	1.9 ± 1.1	
Hypo 30	1.3 ± 0.3	1.7 ± 0.3†	5.2 ± 0.8†,#	5.3 ± 1.1†,#	4.3 ± 1.1†,#	
Hypo 60	1.1 ± 0.3	1.6 ± 0.3‡	8.8 ± 1.1§,‡,#	7.4 ± 1.6‡,#	7.1 ± 1.5§,‡,#	
Systolic diameter (mm)						
Norm 30	4.27 ± 0.07	4.27 ± 0.11	4.17 ± 0.07#	4.25 ± 0.07	4.26 ± 0.07	*P *=* *0.06
Norm 60	4.26 ± 0.06	4.26 ± 0.05	4.02 ± 0.09#	4.33 ± 0.15	4.48 ± 0.17	
Hypo 30	4.37 ± 0.09	4.33 ± 0.12	4.32 ± 0.15	4.47 ± 0.14	4.46 ± 0.12	
Hypo 60	4.26 ± 0.07	4.28 ± 0.06	4.11 ± 0.09#	4.28 ± 0.11	4.40 ± 0.11	
Diastolic diameter (mm)						
Norm 30	4.30 ± 0.08	4.31 ± 0.12	4.19 ± 0.07	4.29 ± 0.06	4.30 ± 0.07	*P *=* *0.08
Norm 60	4.29 ± 0.06	4.30 ± 0.05	4.04 ± 0.10#	4.35 ± 0.15	4.50 ± 0.17	
Hypo 30	4.39 ± 0.10	4.36 ± 0.13	4.35 ± 0.14	4.49 ± 0.13	4.49 ± 0.12†	
Hypo 60	4.28 ± 0.06	4.32 ± 0.05	4.13 ± 0.08	4.30 ± 0.12	4.42 ± 0.10	

Values expressed as mean ± SE. Norm 30, 30% 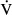
O_2peak_ in normoxia; Norm 60, 60% 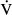
O_2peak_ in normoxia; Hypo 30, 30% 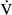
O_2peak_ in hypoxia; Hypo 60, 60% 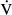
O_2peak_ in hypoxia.

* *P *<* *0.05 Norm 30 versus Norm 60

§ *P *<* *0.05 Hypo 30 versus Hypo 60

† *P *<* *0.05 Norm 30 versus Hypo 30

‡ *P *<* *0.05 Norm 60 versus Hypo 60

# *P *<* *0.05 versus Rest 1 in each trial.

#### Antegrade blood flow and SR

**Figure 1:**
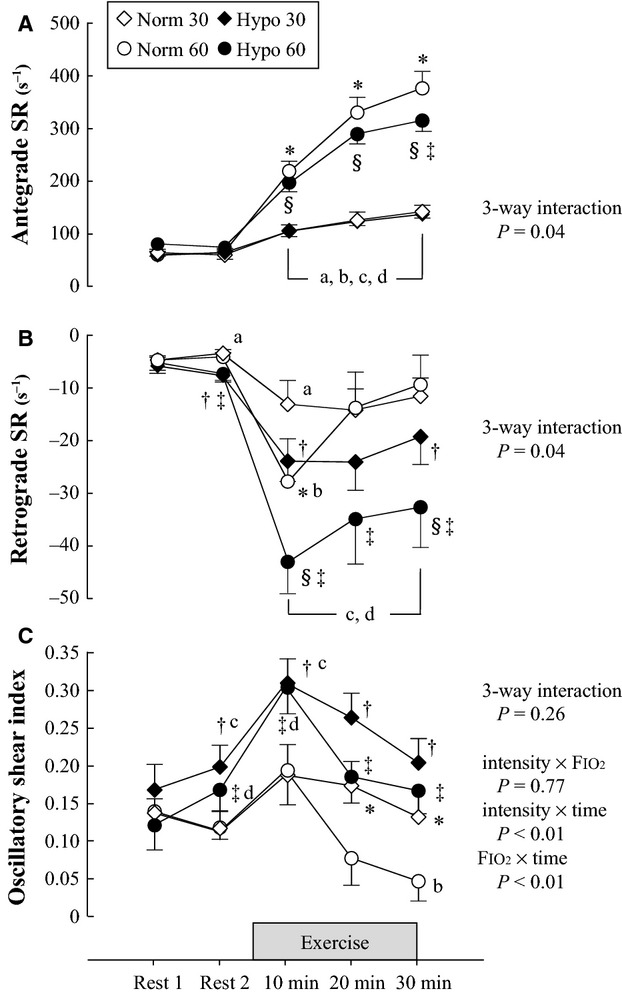
Shear rate (SR) and oscillatory shear index at rest and during exercise under normoxic or hypoxic conditions. (A) antegrade SR, (B) retrograde SR, and (C) oscillatory shear index. Values expressed as mean ± SE. Norm 30, 30% 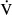
O_2peak_ in normoxia; Norm 60, 60% 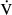
O_2peak_ in normoxia; Hypo 30, 30% 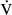
O_2peak_ in hypoxia; Hypo 60, 60% 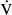
O_2peak_ in hypoxia. *P <* *0.05 Norm 30 versus Norm 60, ^§^*P *<* *0.05 Hypo 30 versus Hypo 60, ^†^*P *<* *0.05 Norm 30 versus Hypo 30, ^‡^*P *<* *0.05 Norm 60 versus Hypo 60, a: *P *<* *0.05 versus Rest 1 in Norm 30, b: *P *<* *0.05 versus Rest 1 in Norm 60, c: *P *<* *0.05 versus Rest 1 in Hypo 30, d: *P *<* *0.05 versus Rest 1 in Hypo 60.

There were significant three-way interactions for antegrade blood flow (*F*_4,28_ = 6.66, Table2) and antegrade SR (*F*_4,28_ = 2.96, Fig.1A). Significant simple interaction effects (intensity × FIO_2_) at 30 min, intensity × time in normoxia and hypoxia, and FIO_2_ × time at 60% 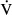
O_2peak_ were observed for antegrade blood flow and SR. There were significant main effects of time on antegrade blood flow and SR, and these variables during submaximal exercise increased from resting values in all trials. Antegrade blood flow and SR showed significant main effects of intensity during exercise in normoxia and hypoxia. These variables were higher during moderate-intensity exercise than during light-intensity exercise under both conditions. Significant main effects of FIO_2_ on antegrade blood flow and SR at 30 min were observed at 60% 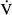
O_2peak_ but not at 30% 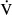
O_2peak._

#### Retrograde blood flow and SR

There were significant three-way interactions for retrograde blood flow and SR (blood flow; *F*_4,28_ = 4.42, SR; *F*_2.29,16.02_ = 3.72, Table2 and Fig.1B). There were significant simple interaction effects (FIO_2_ × time) on retrograde SR at 60% 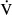
O_2peak_, and for FIO_2_ × time on retrograde blood flow at both exercise intensities. In addition, significant simple interaction effects (intensity × FIO_2_) on retrograde blood flow and SR at 30 min were observed. Main effects of intensity and FIO_2_ on retrograde SR were also significant.

Under normoxia, simple interaction effects (intensity × time) on retrograde blood flow and SR were significant. Main effect of time was observed at 30 and 60% 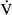
O_2peak_. Retrograde SR and blood flow at both exercise intensities exhibited a biphasic response pattern, with a significant initial increase at 10 min relative to baseline, followed by a decrease. There was a significant main effect of intensity on retrograde SR at 10 min. Retrograde SR at 60% 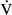
O_2peak_ was significantly greater than that at 30% 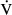
O_2peak_ at 10 min of exercise. However, those differences disappeared with continued exercise. Retrograde blood flow and SR at 20 and 30 min of exercise were unaffected by the difference in exercise intensity under normoxic conditions.

In contrast, there were significant simple interaction effects (intensity × time) on retrograde blood flow and SR under hypoxia. Main effects of time were observed during both hypoxic trials. Retrograde blood flow and SR under hypoxic conditions increased significantly at 10 min of exercise and remained significantly greater than resting values for the duration of the 30-min exercise. Significant main effects of intensity at 10 and 30 min of exercise were observed in hypoxia. Retrograde blood flow and SR were enhanced with increasing exercise intensity, and the differences between light- and moderate-intensity exercise persisted for up to 30 min after exercise in hypoxia.

Figure 3 displays the relationship between changes in skin blood flow and retrograde SR from 10 to 30 min of exercise. A significant negative relationship between changes in skin blood flow and retrograde SR were observed under normoxic conditions (*r *= −0.54, Fig. 3A). In contrast, there was no significant relationship under hypoxic conditions (Fig. 3B).

#### Oscillatory shear index

No significant three-way interactions on oscillatory shear index was observed. There were significant interaction effects (intensity × time and FIO_2_ × time) on oscillatory shear index (Fig.1C). For oscillatory shear index, the main effect of intensity was significant in normoxia, and main effects of FIO_2_ were significant at both exercise intensities. Hypoxia augments oscillatory shear index from Rest 2 to 30 min of light- and moderate-intensity exercise.

## Discussion

The major findings of this study are that (1) retrograde SR in an inactive limb in the early phase of exercise was enhanced with increasing exercise intensity under both normoxic and hypoxic conditions, (2) the differences in retrograde SR between exercise intensities disappeared within 30 min of exercise in normoxia but persisted in hypoxia, and (3) the change in retrograde SR was significantly correlated with the increase in skin blood flow in normoxia but not in hypoxia. We recently demonstrated a progressive increase in retrograde blood flow and SR in brachial artery with increasing exercise intensity during incremental leg cycling (Iwamoto et al. 2012). However, blood flow response during incremental exercise of short duration is not representative of that occurring with prolonged exercise (Simmons et al. 2011a). Aerobic exercise training is typically of long-duration (e.g., over 30 min), constant-load exercise. Therefore, blood flow and SR should be assessed during constant-load exercise under hypoxic conditions. To our knowledge, this is the first study to evaluate the effect of exercise intensity on blood flow and SR in an inactive limb during constant-load exercise in hypoxia.

### Retrograde blood flow and SR under normoxic conditions

It has been suggested that increases in retrograde blood flow and SR in the early phase of exercise is induced by the initial sympathetic vascular constriction and consequent increase in downstream resistance (Blair et al. 1961; Johnson and Rowell 1975; Simmons et al. 2011a). In addition, recent studies have revealed that acute elevations in muscle sympathetic nervous activity are associated with an increase in retrograde SR (Padilla et al. 2010; Thijssen et al. 2014). Increased *α*-adrenergic vasoconstriction (via endogenous norepinephrine release) promotes enhanced retrograde SR, and *α*-adrenergic blockade completely abolishes retrograde SR (Casey et al. 2012). These findings suggest that the change in sympathetic vasomotor outflow is an important modulator of brachial retrograde blood flow and SR during leg cycling exercise. At 10 min of exercise, greater retrograde SR was observed at moderate intensity than that at light intensity, which is consistent with previous studies showing a greater sympathetic vasomotor outflow with increasing exercise intensity under normoxic conditions (Saito et al. 1993; Callister et al. 1994; Ichinose et al. 2008; Katayama et al. 2014a). We need to consider other possible mechanisms responsible for augmenting retrograde SR. First, the change in retrograde SR may be explained by differences in pressure gradients; that is, differences in upstream systemic perfusion pressure relative to the downstream critical closing pressure in the peripheral resistance vessels (Halliwill and Minson 2010). From this, the decrease in upstream pressure during diastole (DBP) could be one reason for the increases in retrograde SR. However, DBP at 10 min of exercise showed no significant change from baseline at both exercise intensities and no difference between 30 and 60% 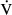
O_2peak_. Second, it has been conjectured that retrograde blood flow and SR are induced by an enhanced elastic rebound during diastole as a consequence of increased SBP and antegrade blood flow during exercise (Thijssen et al. 2009b). At 10 min of moderate-intensity exercise, SBP and antegrade blood flow increased and were higher than those values recorded during light-intensity exercise. These results suggest that higher sympathetic vasoconstrictor tone and elastic rebound resulted in retrograde blood flow and SR that were greater during the early phase of moderate-intensity exercise than during light-intensity exercise under normoxic conditions.

**Figure 2:**
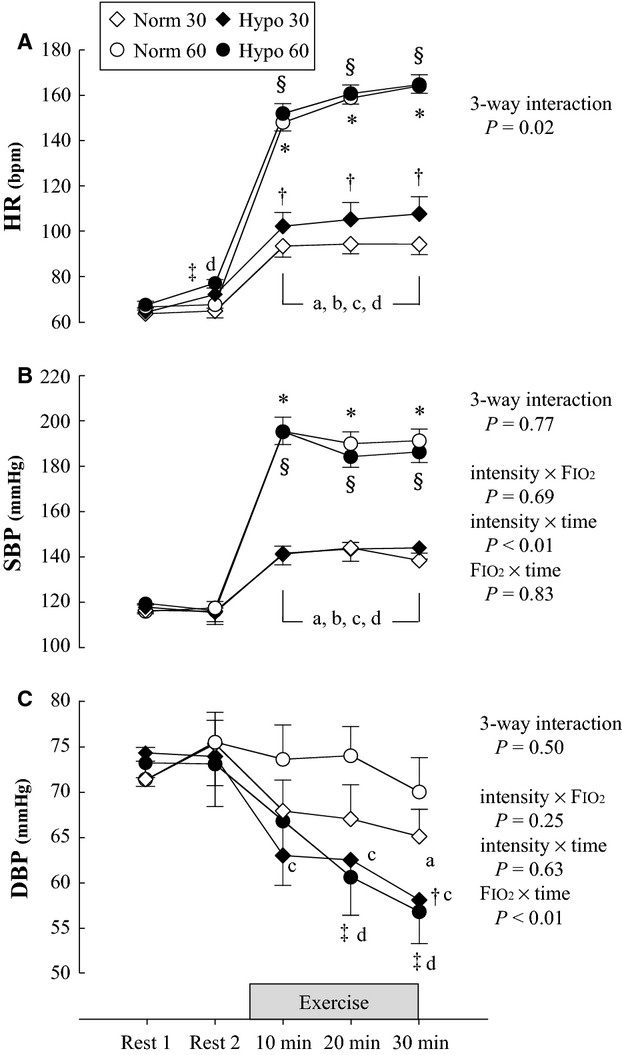
Cardiorespiratory variables at rest and during exercise under normoxic and hypoxic conditions. (A) HR, (B) SBP, and (C) DBP. Values expressed as mean ± SE. SBP, systolic blood pressure; DBP, diastolic blood pressure; HR, heart rate; Norm 30, 30% 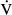
O_2peak_ in normoxia; Norm 60, 60% 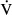
O_2peak_ in normoxia; Hypo 30, 30% 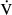
O_2peak_ in hypoxia; Hypo 60, 60% 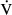
O_2peak_ in hypoxia. *P <* *0.05 Norm 30 versus Norm 60, ^§^*P *<* *0.05 Hypo 30 versus Hypo 60, ^†^*P *<* *0.05 Norm 30 versus Hypo 30, ^‡^*P *<* *0.05 Norm 60 versus Hypo 60, a: *P *<* *0.05 versus Rest 1 in Norm 30, b: *P *<* *0.05 versus Rest 1 in Norm 60, c: *P *<* *0.05 versus Rest 1 in Hypo 30, d: *P *<* *0.05 versus Rest 1 in Hypo 60.

**Figure 3:**
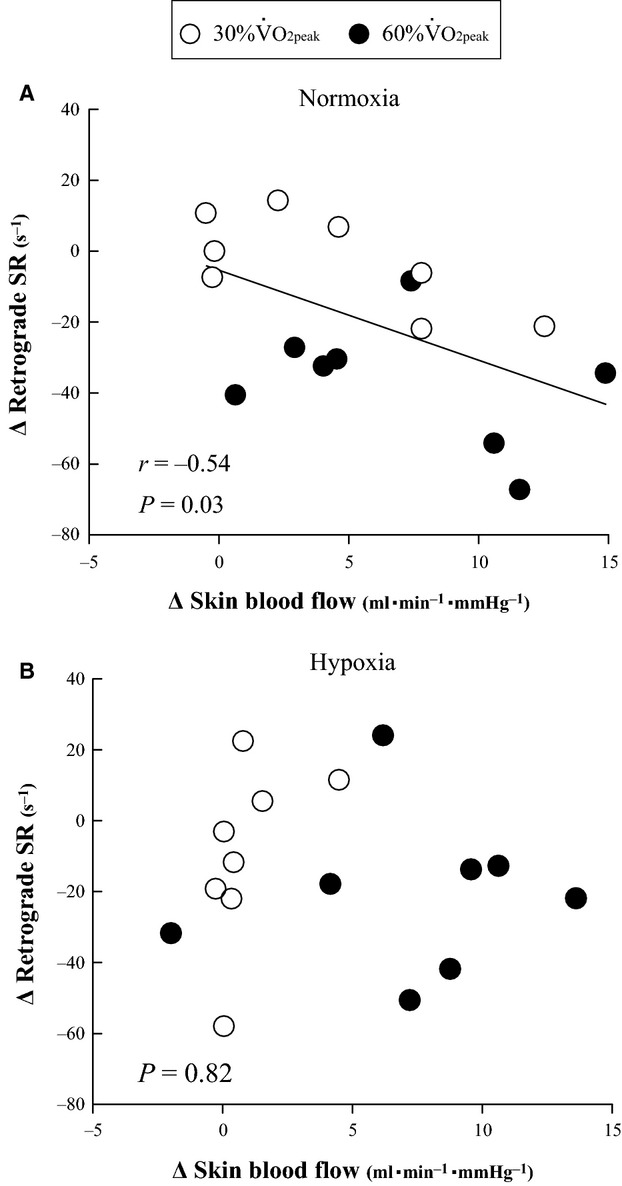
Linear regression analysis of the relationship between changes in skin blood flow and changes in retrograde SR from 10 min to 30 min of exercise under (A) normoxia and (B) hypoxia. SR, shear rate.

As exercise progressed in normoxia, retrograde blood flow and SR returned to baseline levels and the difference between exercise intensities disappeared (Fig.1B and Table2). This result supports our hypothesis. Sympathetic vasomotor outflow was reported to increase gradually during 30 min of leg cycling, even at 40% 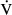
O_2peak_ (Saito et al. 1997). DBP showed a decrease at 30 min of light-intensity exercise in normoxia (Fig.2C). These changes would increase downstream resistance in muscle and decrease upstream pressure, thus leading to enhanced retrograde SR. However, during prolonged exercise in normoxia, the cutaneous circulation is the major effector of forearm vascular resistance (Kamon and Belding 1969; Johnson and Rowell 1975; Simmons et al. 2011a). Skin blood flow increased at both exercise intensities, and that the level to which it increased during 20 min of moderate-intensity exercise was significantly higher than that during light-intensity exercise. The decrease in the magnitude of retrograde SR from 10 to 30 min of exercise was significantly correlated with increased skin blood flow under normoxic conditions (Fig.3A). These results indicate that retrograde SR in normoxia is likely influenced more by increased skin vascular conductance than by other effects, including sympathetic vasomotor outflow and upstream pressure.

### Retrograde blood flow and SR under hypoxic conditions

Hypoxia potentiates exercise-induced sympathetic neural activation (Saito et al. 1991; Seals et al. 1991; Katayama et al. 2011). Our group reported elevated sympathetic vasomotor outflow at 15 min of light-intensity leg cycling in hypoxia, and that this response is enhanced with moderate-intensity cycling (Katayama et al. 2011). The present study demonstrated that SBP and antegrade blood flow increased from resting values under both exercise intensities and that these values were greater at 60% 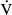
O_2peak_ than at 30% 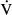
O_2peak_ in hypoxia (Fig.2B and Table2). In contrast, DBP at 10 min of light-intensity exercise in hypoxia significantly decreased from baseline, but there was no difference between light- and moderate-intensity exercise (Fig.2C). This suggests that higher sympathetic vasoconstrictor tone and increased elastic rebound induced retrograde blood flow and SR that were greater in the early phase of exercise at 60% 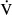
O_2peak_ than at 30% 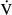
O_2peak_ under hypoxic conditions.

Retrograde blood flow and SR remained elevated above baseline levels throughout 30 min of exercise at both exercise intensities. Retrograde SR was greater at moderate intensity than at light intensity at 30 min of hypoxic exercise. However, skin blood flow and skin vascular conductance increased during 30 min of exercise, and the values were significantly greater at 60% 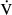
O_2peak_ than that at 30% 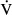
O_2peak_ (Table2). In contrast with normoxic conditions, there was no correlation between the magnitude of retrograde SR and increased skin blood flow from 10 to 30 min of exercise under hypoxic conditions (Fig.3B). These results indicate that the cutaneous circulation is not the major effector of retrograde SR during 30 min of exercise under hypoxic conditions. For muscle vascular conductance, there are no reports evaluating sympathetic vasomotor outflow during prolonged leg cycling at different exercise intensities under hypoxic conditions. However, the increase in sympathetic vasomotor outflow over the initial phase of exercise was reported to be greater at moderate intensity than at light intensity during 15 min of leg cycling (Katayama et al. 2011). Based on these findings, we speculate that sympathetic vasoconstriction at 60% 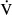
O_2peak_ is greater than that at 30% 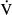
O_2peak_ at 30 min of hypoxic exercise. Considering these results, it is suggested that an increase in downstream muscle vascular resistance induced by a rise in sympathetic vasomotor outflow may be a primary determinant of brachial retrograde SR at 30 min of leg exercise under hypoxic conditions. Consequently, the effect of exercise intensity on retrograde blood flow and SR in the inactive limb during prolonged exercise is greater in hypoxia than that in normoxia.

### Perspective

Increases in retrograde SR and oscillatory shear index have been reported to be a potent stimulus for endothelial dysfunction (Thijssen et al. 2009a, 2014; Schreuder et al. 2014). We found that the elevated brachial retrograde SR induced by increased exercise intensity persisted during 30 min of leg cycling in hypoxia. The negative effect on vascular function is intensified during moderate-intensity exercise in hypoxia. In contrast, enhanced oscillatory shear index returned to baseline values at 30 min of exercise in hypoxia, and there were no differences between intensities. Retrograde SR during exercise at 60% 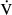
O_2peak_ under hypoxic conditions was greater than under normoxic conditions (Fig.1B). However, there was no significant difference in flow-mediated dilation that is an index of vascular function in the inactive limb following exercise at 60% 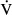
O_2peak_ between normoxic and hypoxic conditions (Katayama et al. [Bibr b28][Bibr b29]). One explanation might be that increased retrograde SR may not always represent an unfavorable stimulus (Braith et al. 2010; Gurovich and Braith 2013). In addition, increases in antegrade shear may prevent any impairment in endothelial function associated with unopposed increases in retrograde flow and shear (Tinken et al. 2009). However, there is a possibility that although increased retrograde SR by hypoxia is a deleterious signal to the endothelium, systemic acute hypoxia and submaximal hypoxic exercise can both induce vasodilation of the conduit arteries (Rowell 1986). No study has examined the effect of exercise intensity on vascular function in the inactive limb under hypoxic conditions, and future studies are needed.

This study suggests that the amount of retrograde blood flow and SR in an inactive limb might be strongly affected by exercise intensity under conditions characterized by low oxygen (e.g. altitude, respiratory disease) and/or elevated sympathetic vasoconstriction outflow. Interestingly, an age-related increase in retrograde SR due to activation of the sympathetic nervous system has been reported (Padilla et al. 2011; Casey et al. 2012). Future studies are needed to examine the impact of exercise intensity on retrograde blood flow and SR under different degrees of hypoxia and for specific diseases.

### Limitations

Potential limitations of the present study should be considered. First, the levels of hypoxia can affect the patterns of blood flow and SR during exercise (Iwamoto et al. 2013). It can also affect inter-individual variability in adaptation to hypoxic environments. Therefore, additional studies are needed to elucidate the changes in blood flow response induced by hypoxic exercise at different intensities combined with varying levels of hypoxia. Second, in the present study, we examined blood flow pattern during light- and moderate-intensity exercise. However, skin circulation increases from moderate- to high-intensity exercise (Simmons et al. 2011b), and sympathetic nerve activity is also enhanced with increasing intensity during leg cycling (Saito et al. 1993; Ichinose et al. 2008; Katayama et al. 2011). These data suggest the contribution of higher exercise intensity in hypoxia to the change in retrograde blood flow (Johnson and Wallace 2012; Birk et al. 2013). Third, we did not measure endothelial function following exercise. Because of this, the present study did not provide insight into the effect of exercise intensities under normoxic and hypoxic conditions on vascular function. In this study, the difference in exercise intensity modulated the blood flow and SR patterns and would affect vascular function. In addition, the increase in exercise intensity produces an increase in oxidative stress, which negatively affects endothelial function (Birk et al. 2013; Dawson et al. 2013; Gurovich et al. 2014). Therefore, consideration should be given not only to blood flow and SR patterns, but also to other factors, including oxidative stress. Finally, indirect DBP measurements during exercise should be considered. DBP measured using the auscultatory method showed a significant reduction from resting value during hypoxic exercise (Fig.2C). Previous studies that used direct arterial measurements also reported decreases in DBP and MAP during exercise under hypoxic conditions (Stenberg et al. 1966; Vogel et al. 1967; Mazzeo et al. 1991; Wolfel et al. 1991, 1998; Wolfel and Levine 2001). These results correspond to the data in the present study. However, it should be noted that the indirect auscultatory method will underestimate internal arterial pressure (BP measured using direct intra-arterial measurement) during exercise, and that this underestimation increases with the workload (Kaijser 1987). Therefore, the effect of DBP on the change in retrograde blood flow and SR during the late phase of exercise should be carefully considered.

## Conclusion

In the present study, we compared blood flow and SR patterns in a brachial artery during leg cycling between light and moderate exercise intensities under normoxic and hypoxic conditions. Retrograde blood flow and SR in the early phase of exercise was enhanced with increasing exercise intensity in both normoxia and hypoxia. As exercise progressed, the increase in retrograde SR dissipated, in part because of cutaneous vasodilation, in normoxia, but persisted in hypoxia. These results suggest that the cutaneous circulation is not the major effector, but rather an increase in downstream vascular resistance may be a primary determinant of brachial retrograde SR at 30 min of leg exercise under hypoxic conditions. We conclude that the differences in exercise intensity affect brachial retrograde blood flow and SR during prolonged exercise under hypoxic conditions.

## Conflict of Interest

None declared.
